# Cefixime and cefixime-clavulanate for screening and confirmation of extended-spectrum beta-lactamases in *Escherichia coli*

**DOI:** 10.1186/s12941-022-00508-4

**Published:** 2022-05-22

**Authors:** Mohammad Al-Tamimi, Hadeel Albalawi, Marwan Shalabi, Jumana Abu-Raideh, Ashraf I. Khasawneh, Farah Alhaj

**Affiliations:** 1 Department of Basic Medical Sciences, Faculty of Medicine, The Hashemite University, Zarqa, Jordan; 2 Department of Pediatrics and Neonatology, Faculty of Medicine, The Hashemite University, Zarqa, Jordan

**Keywords:** *E. coli*, Extended-spectrum beta-lactamases (ESBLs), Cefixime, CTX-M, SHV, TEM

## Abstract

**Introduction:**

Detection of Extended-Spectrum Beta-Lactamases (ESBLs) depends on screening for resistance to certain cephalosporins, confirmation with selective ESBL inhibitors, and ESBL genes detection. New tests are required for accurate ESBL detection.

**Aims:**

To test the ability of cefixime (CFM) and cefixime-amoxicillin/clavulanate (CFM-AMC) as a screening and confirmatory test for ESBL identification.

**Methods:**

246 clinical isolates of *Escherichia coli* were tested by an ESBL screening test, a double-disk synergy test (DDST), a disk replacement test, the Vitek 2 ESBL test, and an ESBL genes test by PCR. CFM ESBL Screening was performed by disk diffusion, while CFM-AMC confirmation was performed by DDST and a disk replacement test.

**Results:**

246 *E. coli* clinical isolates from two referral hospitals were collected over 2 years. The mean age ± standard deviation of patients was 43.8 ± 27.7 years and 76.8% were females. Resistance rates to penicillins, first, second, and third generation cephalosporins, and monobactams were very high at 97%, 84%, 100% and 97%, respectively. ESBL screening was positive in 81.3% of isolates, DDST was positive in 74.8%, disk replacement was positive in 79%, Vitek 2 ESBL test was positive in 67.3%, and ESBL genes were detected in 85.8% of isolates (CTX-M 75%, TEM 42.5%, SHV 4.6%). Compared to genotyping, screening with CFM achieved 87.7% sensitivity and 64.7% specificity. CFM-AMC DDST achieved 75.8% sensitivity and 75.4% specificity, and CFM-AMC disk replacement had 73% sensitivity and 70% specificity.

**Conclusions:**

High prevalence of ESBLs was noted among *E. coli* isolates, dominated by CTX-M genotype. ESBL screening and confirmation using CFM and CFM-AMC is a new and accurate method for ESBLs detection.

## Introduction

*Escherichia coli* is a Gram-negative, rod-shaped, lactose fermenting bacteria of the Enterobacteriaceae family [1]. It is responsible for a wide range of nosocomial and community-acquired infections [1]. Antibiotic resistance in *E. coli* has been increasing worldwide at an alarming rate [2]. Recently, *E. coli* strain*s* resistant to all known antibiotics, including colistin, have been reported [3]. Epidemiological and molecular surveillance, the development of new diagnostic tests, and the discovery of novel therapies for resistant organisms has become a global priority [2, 3].

Antibiotic resistance in *E. coli* is mediated through different mechanisms, including β-lactamase production, porin loss, and efflux pumps [4]. β-lactamase enzymes include extended spectrum β-lactamases (ESBLs), AmpC β-lactamase, and carbapenemase [5]. ESBLs belong to class A and include over 400 enzymes capable of β-lactam drug inactivation. The affected drugs include- penicillins, broad-spectrum cephalosporins, and monobactams, but ESBLs have no effect on carbapenems and cephamycins [6]. ESBLs are generally inhibited by β-lactamase inhibitors like clavulanic acid [7]. SHV and TEM were the most common ESBL genes, but these have recently been surpassed by CTX-M [8].

Multiple large regional studies and recent reviews have highlighted the wide spread of ESBLs in the Middle East region [9–11]. Molecular analysis of ESBL-producing *E. coli* (ESBL-EC) in Jordan was performed in multiple studies with variable rates found [12–15].

Phenotypic confirmation of ESBL production is based on restored susceptibility to third generation cephalosporins with the addition of β-lactamase inhibitors [16, 17]. Phenotypic tests have high rates of errors and can be misleading, so, accordingly, resistance genes detection by polymerase chain reaction (PCR) remains the gold standard [18, 19]. Furthermore, the high false positive rates reported for different phenotypic methods would have a negative impact on patient management [18, 19]. Development of a new and reliable phenotypic test for accurate detection of ESBL-producing strains is required. In a previous study, cefixime and amoxicillin/clavulanate was evaluated as an effective oral combination therapy for treating ESBL-EC, demonstrating a strong *in vitro* synergistic effect [20]. The aims of this study are an in-depth phenotypic and molecular characterization of β-Lactamase-producing *E. coli* isolates form multiple referral centers in Jordan and an evaluation of the ability of cefixime and amoxicillin/clavulanate to detect ESBL compared to standard and molecular methods.

## Materials and methods

### Patients and bacterial isolates

A total of 246 clinical isolates were included from patients with *E. coli* infections from Prince Hamzah Hospital and Islamic Hospital in Amman, Jordan, from October 2017 to December 2019. All isolates were collected after obtaining voluntary consent and ethical approval. Isolates were identified by standard microbiological procedures including culture on MacConkey agar, Gram stain, and manual biochemical tests, including citrate, indole, methyl-red, and voges-proskauer tests. Furthermore, species confirmation was carried out using the Vitek 2 compact system, using a Gram-negative identification card (BioMerieux, France).

### Antibiotic susceptibility tests

Antibiotic susceptibility testing of isolates was performed using a standard disk diffusion test and the Vitek 2 compact system, using a Gram-negative antibiotic susceptibility card (AST GN69, BioMérieux, France). The following antibiotics were tested: ampicillin, amoxicillin/clavulanate, piperacillin/tazobactam, cephalosporins (cefazolin, cefuroxime, cefoxitin, ceftriaxone, ceftazidime, cefotaxime, cefixime, cefpodoxime, cefditoren, cefepime), carbapenems (imipenem, meropenem, ertapenem), monobactams (aztreonam), aminoglycosides (tigecycline, gentamicin, amikacin), quinolones and fluoroquinolones (nalidixic acid, norfloxacin, ciprofloxacin, levofloxacin), folate pathway antagonists (trimethoprim/sulfamethoxazole) and nitrofurans (nitrofurantoin). Zones of inhibition were interpreted according to the latest recommendations of CLSI [17].

### Screening and confirmation tests for ESBL enzymes

An ESBL screening test was performed by disk diffusion, while ESBL confirmation was tested by double-disk synergy testing and disk replacement testing using cefpodoxime (10 µg), ceftazidime (30 µg), and cefotaxime (30 µg) with or without amoxicillin/clavulanate following CLSI criteria [17, 20]. Furthermore, an automated Vitek 2 ESBL confirmation test based on simultaneous assessment of the inhibitory effects of cefepime, cefotaxime, and ceftazidime, alone and in the presence of clavulanic acid, were applied (NO45 card, BioMérieux, France).

ESBL Screening with cefixime (5 µg) was performed using a disk diffusion method and interpreted according to the last recommendation by CSLI (resistant ˂ 15 mm, intermediate = 16–18 mm, sensitive ≥ 19 mm) [17], while cefixime confirmation with amoxicillin/clavulanate (20/ 10 µg) was detected using the double-disk synergy test at 20 mm distance and a disk replacement test similar to other recommended cephalosporins [20].

### Molecular characterization

DNA extraction was performed following the procedure recommended by the manufacturer (Qiagen, Hilden, Germany). ESBL encoding genes (CTX-M, TEM, and SHV) were detected by uniplex PCR using specific and universal primers and protocols described previously that detect different variants of each gene [21, 22]. All primers were obtained from University of Science and Technology, Jordan. PCR products were electrophoresed on 2% agarose gel stained with Ethidium bromide and visualized under UV transillumination.

### Statistical analysis

Statistical analysis was performed using SPSS version 24. A P value less than or equal 0.5 was considered statistically significant. Descriptive analysis was used to calculate the prevalence of variables. The correlation between phenotypic and genotypic methods was tested by Chi-square or the Fisher exact test. The crosstab was used to calculate the sensitivity (the proportion of true positives tests out of all patients) and specificity (the percentage of true negatives out of all patients) of each method.

## Results

### Demographic characteristics of patients

A total of 246 *E. coli* clinical isolates were collected from two referral hospitals over 2 years including126 isolates from Islamic Hospital and 120 isolates from Prince Hamzah Hospital. The mean age of patients was 43.82 ± 27.7 years. One hundred eighty-two samples (76.8%) were obtained from females and 56 (23.5%) were obtained from males. Urine samples were the most common sources of isolates (87.2%), while most recruited patients were from the pediatric department (Table 1).

**Table 1:** Characterization of study participants with *E. coli* infections (n = 246)

Variable	Category	Percentage of *E. coli *(%)
Hospital	Islamic Hospital	51.2
	Prince Hamzah Hospital	48.8
Age (years)	≤ 20	25.2
	21 to 40.9	21.1
	41 to 60.9	17
	61 to 80.9	26.6
	> 80.9	10.1
Gender	Male	23.2
	Female	76.8
Department	ICU	14.6
	Emergency	17.5
	Medicine	8
	Pediatric	21.9
	Surgery	10.9
	Urology	15.3
	Others	11.7
Type of samples	Blood	2.3
	Urine	87.2
	Sputum	2.3
	Wound	4.1
	Others	4.1

### Antibiotic susceptibility pattern of isolates

**Fig. 1:**
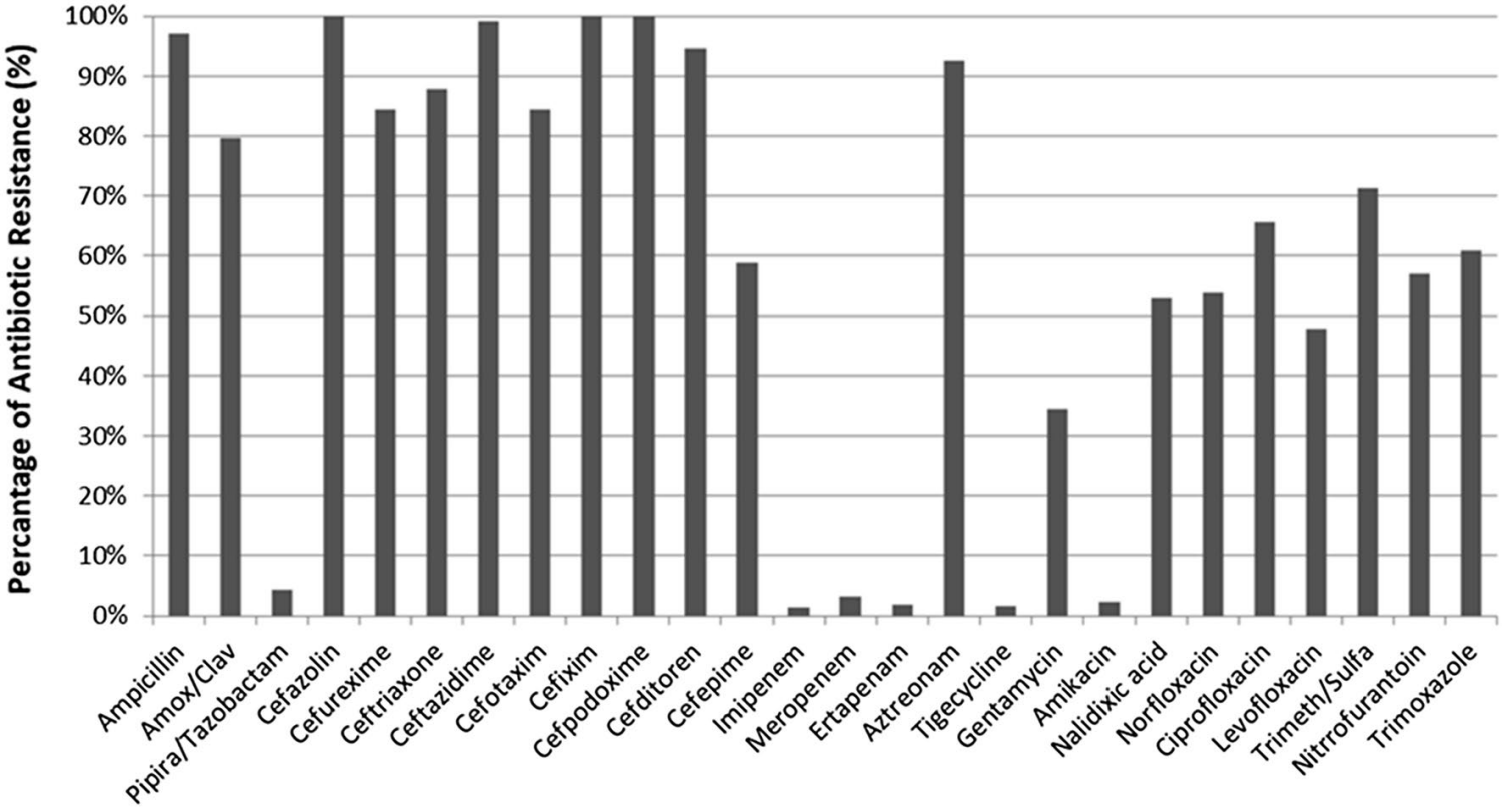
Antibiotic resistance pattern for *E. coli* isolates by disk diffusion method (n = 246)

Resistance rates to penicillins, first, second, and third generation cephalosporins, and monobactams were very high at 97%, 84%, 100% and 97%, respectively. A high resistant rate was observed for amoxicillin/clavulanate (79.6%) relative to the lower resistance rate for piperacillin/tazobactam (4.3%). Furthermore, a high resistance rate was noted for Trimethoprim/sulfamethoxazole (72%), while the resistance rate for aminoglycosides was less than 50% and for quinolones, fluoroquinolones and nitrofurans was above 50%. Only 5% of isolates were resistant to carbapenems (Fig. 1).

### Phenotypic and genotypic detection of ESBLs

The ESBL screening test was positive in 81.3% (200/246) of *E. coli* isolates, including 81.5% that were resistant to cefotaxime (CTX), 80.2% that were resistant to cefpodoxime (CPD), and 76.5% that were resistant to ceftazidime (CAZ). ESBL confirmation by DDST was positive in 74.8% (181/242) of isolates. CTX yielded the highest synergistic activity with clavulanic acid (69.9%), followed by CAZ (58.3%) and CPD (56.4%). About 79% of isolates were positive by the disk replacement method, including 74.3% that were positive with CTX, 65% with CAZ, and 58.8% with CPD. 67.3% of isolates were positive by the Vitek 2 ESBL detection card (Table 2).

**Table 2:** Phenotypic tests for detection of *E. coli*-producing ESBL (n = 246)

`		Percentage of positive (%)	Percentage of negative (%)
ESBL screening test	CTX or CPD or CAZ	81.3	18.7
	CTX	81.5	18.5
	CPD	80.2	19.8
	CAZ	76.5	23.5
	CFM	78.9	21.1
Double-disk synergy test	CTX or CPD or CAZ	74.8	25.2
	CTX	69.9	30.1
	CAZ	58.3	41.7
	CPD	56.4	43.6
	CFM	66.5	33.5
Disk replacement test	CTX or CPD or CAZ	79	21
	CTX	74.3	25.7
	CAZ	65	35
	CPD	58.8	41.2
	CFM	68.7	31.3
Vitek 2 ESBL test		67.3	32.7

**Fig. 2:**
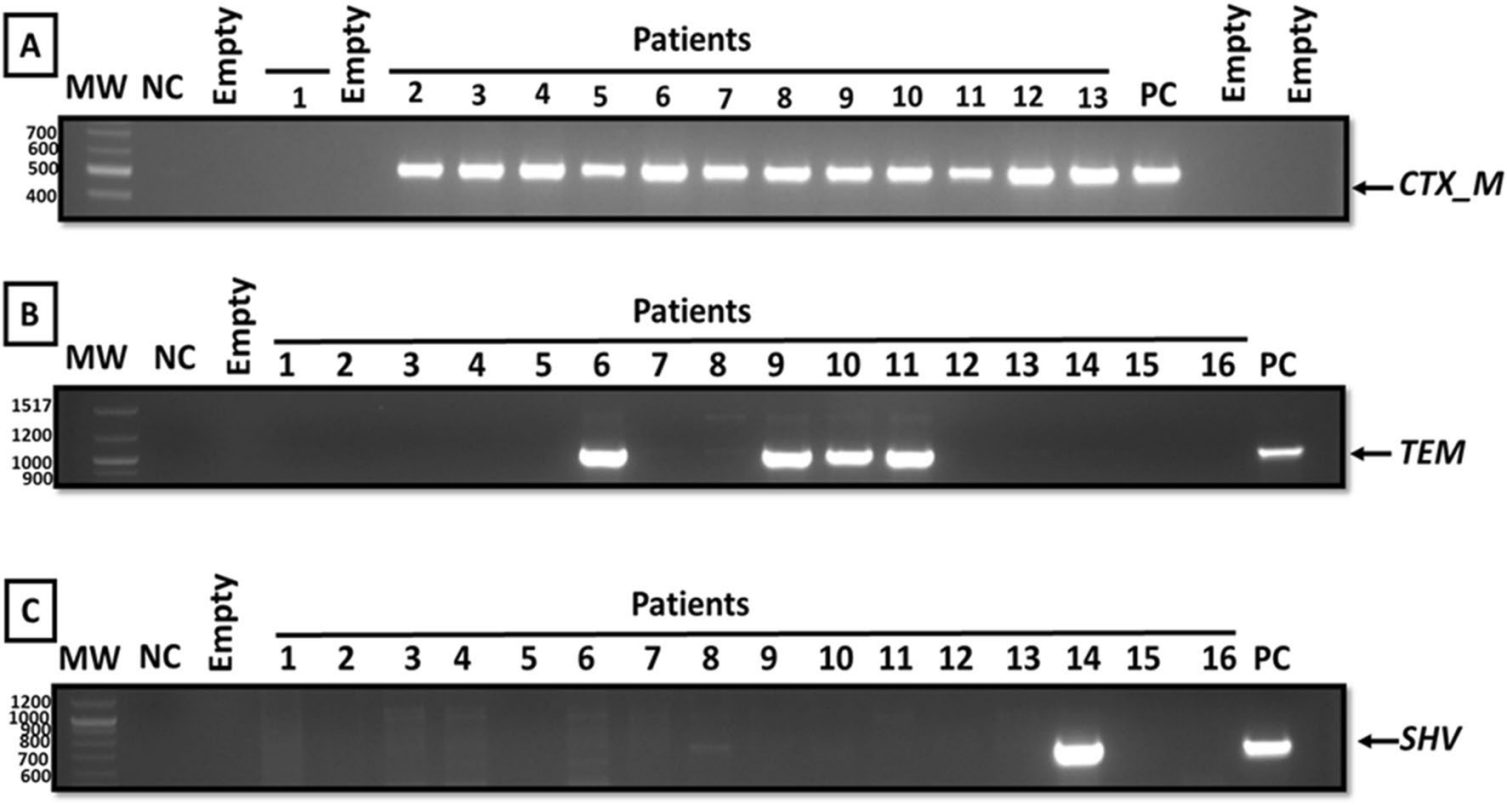
Analysis of PCR product by gel electrophoresis stained with ethidium bromide showing a band at around 550 base pairs for CTX-M **(A)**, a band at around 1080 base pairs for TEM gene **(B)**, and a band at around 795 base pairs for SHV gene **(C)**. **MW**: Molecular weight ladder of 100 bp, **NC**: Negative control, **Empty**: empty control, and **PC**: Positive control (CTX-M-, TEM- and SHV- positive-*E. coli* NCTC strains)

ESBL genes were detected in 85.8% of isolates. The CTX-M gene was predominantly detected in 75% of isolates, followed by the TEM gene in 42.5% of isolates and the SHV gene in 4.6% (Fig. 2). 32.5% of isolates had CTX-M and TEM genes simultaneously, while 3.3% of isolates had CTX-M and SHV genes and 1.6% had TEM and SHV genes. The three ESBL genes were detected simultaneously in only 1.2% of the isolates (Table 3). Prevalence and frequencies of ESBL genes among isolates were not affected by age, gender, sample types or sources, or hospital department (P > 0.05) (Data not shown).

**Table 3:** Distribution of ESBL among *E. coli* strains (n = 246)

ESBL genes	Percentage of positive (%)
CTX–M or TEM or SHV	85.8
CTX–M	75
TEM	42.5
SHV	4.6
CTX–M + TEM	32.5
CTX–M + SHV	3.3
TEM + SHV	1.6
CTX–M + TEM + SHV	1.2

### Performance of phenotypic tests for detection of ESBL genes

A molecular method was used as the reference method to evaluate the performance of phenotypic testing for ESBL detection. For the ESBL screening test, the overall sensitivity and specificity were 89.8% and 61.8%. The sensitivity and specificity for CPD were 88.7% and 63.6%, CTX were 90.1% and 62.1%, and CAZ were 84.5% and 64.7%. For the confirmation test, the overall sensitivity and specificity of DDST were 84.3% and 71.9%, including CTX-AMC at 78.3% and 72%, CAZ-AMC at 64.2% and 71.9%, and CPD-AMC at 63.5% and 75.8%, respectively. Disk replacement testing showed a higher sensitivity rate compared to DDST at 85.5% with a lower specificity rate at 64%. The sensitivity and specificity of the disk replacement method using CTX-AMC were 80.5% and 65.2%, CAZ-AMC were 70% and 70.4%, and CPD-AMC were 63.1% and 70.8%, respectively. Vitek 2 ESBL confirmation testing achieved the lowest sensitivity rate at 76.1% but the highest specificity rate at 100% compared to the other tests (Table 4).

**Table 4:** The sensitivity and specificity rates of four phenotypic ESBL detection methods compared to the reference method of genotyping

		Sensitivity (%)	Specificity (%)
ESBL screening test	CPD or CTX or CAZ	89.8	61.8
	CPD	88.7	63.6
	CTX	90.1	62.1
	CAZ	84.5	64.7
	CFM	87.7	64.7
Double-disk synergy test	CPD or CTX or CAZ	84.3	71.9
	CPD	63.5	75.8
	CTX	78.3	72
	CAZ	64.2	71.9
	CFM	75.4	75.8
Disk replacement test	CPD or CTX or CAZ	85.5	64
	CPD	63.1	70.8
	CTX	80.5	65.2
	CAZ	70.	70.4
	CFM	73	70
Vitek 2 ESBL test		67.1	100

### CFM for screening and confirmation of ESBL phenotype

Of the total number of isolates, 75.2, 3.7 and 21.1% were resistant, intermediate, and sensitive to CFM, respectively. Using CFM with DDST was positive in 66.5%, and in 68.7% with the disk replacement method. Compared to genotyping, screening with CFM achieved 87.7% sensitivity and 64.7% specificity. CFM-AMC confirmation by double-disk synergy test achieved 75.4% sensitivity and 75.8% specificity rates, while the sensitivity and specificity of CFM-AMC using the disk replacement method were 73 and 70% (Table 4). Increasing the cut-off point of CFM resistance increased the sensitivity of the distance without affecting its specificity (Table 5). Similarly, using 1 mm of synergy distance for CFM-AMC in DDST enhanced the sensitivity rate without affecting specificity (Table 5).

**Table 5:** The sensitivity and specificity rates of CFM for screening of ESBL using disk diffusion method at deferent breakpoints and for CFM-AMC DDST at different synergy distances

Test	Breakpoint	Sensitivity (%)	Specificity (%)
ESBL screening	≤ 14 mm	81.3	64.7
	≤ 15 mm	83.3	64.7
	≤ 16 mm	84.7	64.7
	≤ 17 mm	87.2	64.7
	≤ 18 mm	87.8	64.7
	≤ 19 mm	88.2	64.7
	≤ 20 mm	88.7	64.7
	≤ 21 mm	89.2	64.7
Double-disk synergy test	Distance	Sensitivity (%)	Specificity (%)
	≥ 1 mm	75.4	75.8
	≥ 3 mm	71.3	75.8
	≥ 5 mm	63.3	75.8

## Discussion

In this study, a high prevalence of ESBL-EC (85.8%) was documented by presence of at least one known ESBL gene among hospitalized patients from two centers. This agrees with most previous studies in Jordan which indicated a dramatic increase in ESBL-EC in recent years [12, 15, 23–26]. The high prevalence of ESBL-EC isolates is alarming and would limit treatment options for patients. These isolates were susceptible to aminoglycosides and carbapenem drugs, which are available mostly as parenteral drugs and would require hospitalization with all of its clinical, social, and economic impacts. The antibiotic susceptibility pattern of *E. coli* isolates in this study indicated over 50% resistance to all antibiotic classes except aminoglycosides and carbapenems. This is similar to other studies [15, 20, 23, 27].

Molecular methods were used as a reference method to evaluate the performance of different phenotypic ESBL tests. In this study, four phenotypic methods were performed on all *E. coli* isolates. The ESBL screening test had the highest sensitivity (89.8%) but the lowest specificity (61.8%) rate. Confirmatory tests, including the disk replacement test, had an 85.5% sensitivity rate and 64% specificity rate, DDST had an 84.3% sensitivity rate and 71.9% specificity rate, while automated Vitek 2 ESBL test had the lowest sensitivity rate at 67.1% but the highest specificity rate of 100%. These results are comparable to other studies, which indicates variable but high rates of false positive results of the different phenotypic methods compared to genotyping (broth microdilution, DDST, disk replacement test, and Vitek 2 ESBL test) [18–20, 28, 29].

The CTX-M gene was the most common ESBL gene isolated in this study, which is similar to other studies [12, 15, 23]. The TEM gene accounted for 42.5% of ESBL genes, which is higher than previously reported by Nimri et al. (23.6%) [12] and lower than the reported percentage (69%) by another study [24]. The SHV gene accounted for 4.6% while previous studies reported 0%, 1%, 14.3% and 30.6%, respectively [12, 15, 23, 24].

About 3.8% of *E. coli* isolates were positive for an ESBL phenotypic confirmation test with absence of detectable ESBL genes (CTX-M, TEM, and SHV), possibly due to the presence of other less common ESBL genes [7]. Furthermore, 8.8% of isolates were negative by all ESBL confirmation tests but had one detectable ESBL gene, indicating a false negative result [30] or the presence of unexpressed ESBL genes similar to susceptible *Klebsiella pneumoniae* [30] and *E. coli* isolates [31]. Interestingly, 15 (6.3%) of isolates were positive by a screening test andnegative by confirmation tests even though they had detectable ESBL genes (11/15 had CTX-M gen and 7/15 had TEM gene). These strains mostly possess AmpC beta-lactamases that masked the inhibitory effect of the ESBL inhibitor clavulanic acid [5].

Among different antibiotics used for ESBL screening, CTX was the most sensitive while CAZ was the most specific. CFM achieved high sensitivity and specificity rates that were equal to or better than other antibiotics used for ESBL screening. Furthermore, CFM-AMC was superior compared to other antibiotics in DDST and disk replacement tests. Increasing the cut-off point of CFM resistance in disk diffusion and using 1 mm of synergy distance for CFM-AMC in DDST enhanced the sensitivity rate without affecting the specificity rate.

## Conclusions

A high prevalence of ESBL production was noted among *E. coli* isolates from two referral centers in Jordan. CTX-M was the most prevalent ESBL gene (75%), followed by TEM at 42.5% and SHV at only 4.6%. The ESBL Screening test achieved the highest sensitivity but the lowest specificity rates of all phenotypic tests. DDST and disk replacement testing were comparable in their sensitivity rates while DDST was superior in specificity rate. Vitek ESBL testing had the lowest sensitivity but the highest specificity rate. CFM was equal or superior to other antibiotics used for ESBL screening while CFM-AMC testing was superior compared to other antibiotics in DDST and disk replacement testing. Using CFM and CFM-AMC disks for detection of ESBLs provides a new, simple, and accurate method.
